# Newborn screening for congenital hypothyroidism: improvement in short-term follow-up by audit and monitoring

**DOI:** 10.1186/s13104-020-05400-y

**Published:** 2020-12-14

**Authors:** Hafsa Majid, Sibtain Ahmed, Imran Siddiqui, Khadija Humayun, Hussain Karimi, Aysha Habib Khan

**Affiliations:** 1 Department of Pathology and Laboratory Medicine, Aga Khan University, Stadium Road, P.O. Box 3500, Karachi, 74800 Pakistan; 2 Department of Pediatrics and Child Health, Aga Khan University, Stadium Road, P.O. Box 3500, Karachi, 74800 Pakistan; 3 Jinnah Sindh Medical University, Karachi, Pakistan

**Keywords:** Congenital hypothyroidism, Thyroid-stimulating hormones, Newborn screening, Critical results

## Abstract

**Objective:**

Newborn screening for congenital hypothyroidism (CH) at our hospital during this study was by measurement of thyroid stimulating hormone (TSH) in serum samples. This audit was conducted over a 2 year period, to determine the compliance of reporting of results greater than the screening cutoffs for serum TSH. Gaps of non-compliance were identified, and re-audit was undertaken after the corrective actions were taken.

**Results:**

The critical limit was defined as serum TSH (≥ 20 µIU/ml) following consultation with a pediatric endocrinologist. All results above this limit were reported urgently to physicians. During the audit period, 27,407 tests were performed, 0.7% had a value of ≥ 20 µIU/ml, of those only 62% were reported to the general paediatrician or neonatologist. Reasons for not reporting results included non-availability of contact information, lack of policy awareness by technologists, critical results not highlighted on the computer display, and absence of regular monitoring. Corrective measures were taken, and re-audit was done. During the re-audit period, a total of 22,985 tests was performed, 0.6% had a value of ≥ 20 µIU/ml. Of these, 77% were reported to the general paediatrician or neonatologist. Critical result reporting was improved after the audit, and further enhanced the laboratory service of CH screening.

## Introduction

Newborn screening (NBS) programs are considered the most extensive preventive medicine system and a vital element of public health. The newborns are tested for specific disorders, not symptomatically present at the time of birth, but if remain untreated can permanently impact the health of the baby. The main goal of NBS programs is to reduce morbidity and mortality and improve the health outcomes of newborns. Congenital hypothyroidism (CH) is a disorder which if screened for in every newborn has a high benefit-to-risk ratio, as a cost-effective treatment for it is available, it can lead to mental retardation if not identified and treated within first few weeks of birth.

A key-issue in post-analytical quality is represented by the effectiveness of communication of laboratory data, particularly communication of critical test results [1]. The idea of critical values reporting to physicians was first presented by a pathologist, George D. Lundberg, in 1990. It was felt that communicating abnormally high or low laboratory results to the treating physicians can help in initiating treatment promptly in life-threatening situations. Later, critical values reporting received more focused attention by regulatory authorities and agencies, including Clinical Laboratory Improvement Amendments of 1988 (CLIA), the Joint Commission (JC) and the College of American Pathologists (CAP) [2, 3]. The JC National Patient Safety Goals of 2005, amended in 2013, included a requirement of regularly evaluating the timeliness of communication and appropriate actions to be taken to improve it. Communicating critical test results was also included in patient safety solutions of the World Alliance for Patient Safety and World Health Organization (WHO) Collaborating Centre for Patient Safety Solutions updated in 2008 by the WHO’s International Steering Committee [1, 4].

Laboratories are responsible for testing, reporting critical results to health care providers, tracking, and improving the timeliness of reporting [5]. Regulatory authorities have put forth safe practice recommendations regarding issues related to defining cutoffs for critical results and their communication [6]. But there exists no consensus on the choice of analytes to be included in the list of critical results, and clinical laboratories follow both recommendations of scientific societies and clinician’s opinions in their institutions [7]. Secondly, there is no consensus on cutoffs for routine biochemical analytes. Laboratories have to develop their critical results list, define cutoffs, do regular audits, update this list and cutoffs according to recent guidelines. However, for newborn screening; it is recommended that laboratories must determine screening cutoffs balancing true and false positives, and determine whether a particular result must be reported urgently or not urgently, e.g. in CH screening a probable disease result, e.g. over 20 µIU/ml serum would be reported urgently, e.g. by phone for recommended action of the clinical referral or diagnostic testing.

One screening test that is critical, and requires immediate clinical action is thyroid-stimulating hormone (TSH), performed in neonates for screening and confirmation of congenital hypothyroidism (CH). Timely reporting of critical values for TSH leads to early treatment, preventing mental retardation, and is therefore vital to patient safety [8]. According to the American Academy of Pediatrics (AAP) guidelines, nearly 10% of infants with confirmed CH have TSH values between 20 and 40 mIU/l, where conversion factor for TSH from mU/l to µIU/ml is 1. It is advisable that in cases with screening test TSH concentrations between 20 and 40 µIU/ml, serum-free thyroxine (FT4) and repeat TSH should be analyzed [8].

The estimated incidence of CH is 1 in 1000–1600 live births in Pakistan, which is higher than the reported incidence in western countries [9, 10]. Guidelines recommend newborn screening for CH should be performed between 2 and 4 days of age (48–72 h of birth) by analyzing TSH (generally on dried blood spot samples), and confirmation is done by performing serum TSH and FT4 [11]. According to AAP and American Thyroid Association (ATA) guidelines for newborn screening and therapy for CH newborn screening test results must be communicated rapidly back to the general paediatrician or neonatologist, who is responsible for the follow-up of the newborn, for rapid diagnostic testing and decision making regarding patient management [12]. Prompt reporting of high TSH to the physician will ensure that treatment is instituted earlier. So this audit was planned to evaluate the compliance of reporting of results greater than the screening cutoffs for serum TSH and develop a mechanism for short-term follow-up.

## Main text

### Methods

An audit was conducted at Section of Chemical Pathology, Department of Pathology and Microbiology at Aga Khan University (AKU). The audit was performed from January 2015 to December 2016, followed by gap analysis, implementation of corrective/preventive measures, and re-audit in January 2018 to December 2019. At our centre, infants are discharged after 48 h, and samples are taken just before discharge. Serum TSH is analyzed at an automated analyzer ADVIA Centaur (Siemens Diagnostics, US) using a chemiluminescence immunoassay technique.

The critical result reporting policy for TSH was defined in consensus with the clinicians, according to the published guidelines for CH screening and diagnosis by AAP and ATA. Only one level of TSH, i.e. ≥ 20 µIU/ml, was taken as the critical limit. The communication of critical results procedure was developed as per standards defined by the JC and CAP. In the case of inpatients, the attending physician was notified while for outside referrals, parents/guardian were communicated the critical results. The goal was that 100 per cent of critical results should be notified to the concerned person, within 24 h of result availability. The results reported, the technologist informing and parent/physician informed, date and time of result reporting, results of TSH, and results read back by the person briefed were documented. The results which were not critical were reported as per the routine laboratory practice.

Data were analyzed by Microsoft Excel 2010. Frequencies of serum TSH tests performed, total critical results analyzed and communicated, and reasons for non-communication were investigated.

### Results

A total of 27,407 neonates were screened for CH by measurement of TSH over 2 years, from 2015 to 2016. The Median (Q3 − Q1) age of neonates was 3 days (2–4). During the audit period, 0.7% (n = 182) of critical results were obtained. The distribution of results appropriately reported not reported, and reasons for not reporting are shown in Table 1.

**Table 1:** Findings of audit and re-audit on status of critical results reported to General Pediatricians and Neonatologists

Cut off (µIU/ml)	N	Results reported/communicated	Results not reported/communicated			Repeat TSH n (%)	FT4 done n (%)
			Call not received	Contact not available	Other reason		
Audit (Jan 2015–Dec 2016) n = 27,407							
≥ 20	182	113 (62%)	17 (9%)	15 (8%)	37 (20%)	50 (23%)	31 (17%)
Re-Audit (Jan 2018–Dec 2019) n = 22,985							
≥ 20	139	107 (77%)	8 (6%)	13 (9.4%)	11(8%)	75 (54%)	60 (43%)

During the audit period, only 23% (n = 50) and 17% (n = 31) patients were retested for serum TSH (23%) and FT4 (17%), respectively (Table 1). After the audit, the project team met to discuss the findings, identify gaps, suggest corrective or preventive actions, and devise a strategy to implement these actions, shown in Table 2.

**Table 2:** Gaps identified after audit and correct or preventive actions taken

S#	Gap identified	Corrective/preventive action taken
1	Unavailability of contacts of mostly outside referral patients	Medical receptionists and personnel entering information in the medical records were reminded to take patient contact numbers while ordering the TSH test
2	Technologists lack critical results reporting policy knowledge	A competency assessment of technologists was performed to evaluate knowledge of the critical results reporting policy Knowledge of the policy was made part of the technologist competency assessment form for serum TSH analysis
3	Critical results for TSH were not highlighted on a computer display	Critical results of TSH were highlighted and in a different colour in the integrated laboratory management system
4	No monitoring of critical results reported	The critical result reported was made a quality indicator with daily monitoring The goal was set as 100% critical results reported

Re-audit was performed from January 2018 to December 2019 after the implementation of corrective actions. During re-audit period, 22,985 neonates were tested for TSH with a median age of 3 days (2–4), and 0.6% (n = 139) critical results were obtained. The distribution of results reported, patients retested for TSH and FT4 is shown in Table 1 which shows improvement in newborns retested for TSH (54% vs 23%) and FT4 (43% vs 17%) during re-audit period, compared to the audit period.

### Discussion

Timely reporting of critical newborn screening results to the physicians is crucial for patient + 6 safety and prompt initiation of, thyroxine treatment to avoid irreversible mental retardation. In the audit period, 0.7% (n = 182) patients had critical TSH levels, and only two-thirds of these were reported appropriately.

Guidelines also recommend repeating TSH and perform FT4 test before initiating CH treatment, so we also evaluated the number of repeat TSH, and FT4 performed on patients with critical results. In the present study, only 54% of patients were retested for serum TSH, and 43% were also tested for serum FT4 during the re-audit period. Although the number of patients retested was increased compared to the audit period, however, it was lower than the previously reported audits. An audit done by Jones JH on the improvements in screening performance of a Scottish newborn screening program for CH, observed that only 82% of the newborns notified of high TSH results were resampled for TSH analysis [13]. The reason for this difference could be that patients were evaluated and tested at another laboratory. Another reason can be that AAP guidelines suggest that every newborn with serum TSH ≥ 40uIU/ml should immediately be initiated on thyroxine treatment and simultaneously a repeat sample should be sent for TSH and FT4 analysis for the confirmation of CH. In contrast, only 10% of the newborns with serum TSH levels between 20 and 40 µIU/ml develop confirmed CH. When using a higher cutoff, i.e. serum TSH of ≥ 40 µIU/ml for the recall of patients, physicians will be more inclined towards retesting for both TSH and FT4 (1 µIU/ml = 1 mU/l).

Gap analysis revealed that the most common reason for non-communication of critical results was non-availability of the contact information or phone calls not received by physicians or patients’ parents/guardians. A similar survey was done in the Italian population reporting 7 years’ experience from the screening centre in the Lombardy region, Italy. The authors reported that the main reason for the non-communication of critical results was the relocation of families and lack of contact information. [14]. Another study from the Hospital for Sick Children in Toronto, Ontario, reported that potential reasons for non-communication of results were service-related commitments of staff responsible for informing and attempting to find ordering physicians via informal channels [15]. In the present study, the technologist responsible for analysis and communicating or notifying critical serum TSH results were unable to do so due to their high workload. Since there was no monitoring system in place, there were delays in reporting the critical result.

**Fig. 1:**
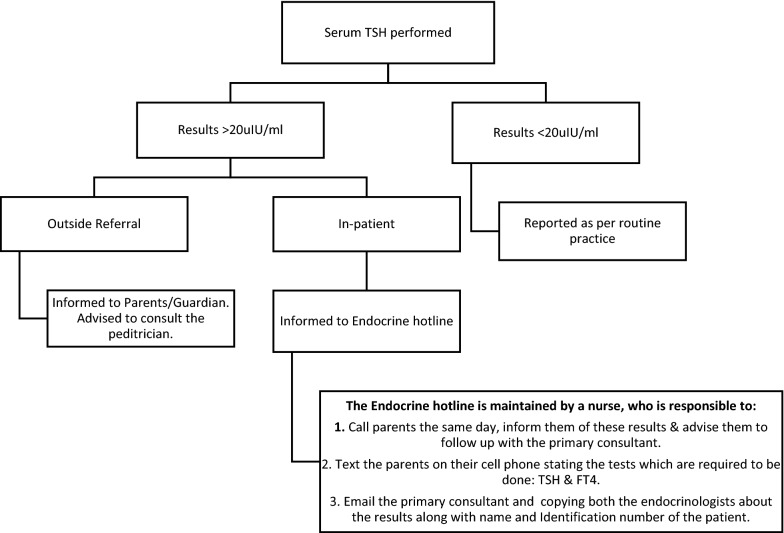
Communication pathway for serum TSH critical results reporting

After the re-audit, the audit team gave the following recommendation:

- Communication pathway: a communication pathway be developed (Fig. 1), as the most common reason for non-communication was the unavailability of contact information or phone not received. In a new pathway, all results of inpatients with levels ≥ 20 µIU/ml should be informed to a pediatric endocrine nurse available at the endocrine hotline. The nurse notifies these results to the patients’ general paediatrician or neonatologist, and pediatric endocrinologists. She also calls and advice the patient to perform confirmatory testing and arranges a follow-up consultation.
- For outside referrals, parents are notified of the results and advised to consult the patients’ general paediatrician or neonatologist, shown in Fig. 1.
- The collecting laboratory should ensure that contact information is included on all serum TSH request forms.
- Communications are monitored and audited regularly: the audit team further suggested that the percentage of subjects notified with serum TSH ≥ 20 µIU/ml should be made a quality indicator and regularly monitored, as well as audited periodically.
- Interpretative comments on reports: after the re-audit period, only 30–55% of patients were retested for TSH and FT4. To increase the proportion of babies receiving repeat testing, a comment can be added to reports of critical TSH levels recommending to repeat TSH and perform FT4 testing and follow up with paediatrician advised for further management. This comment was added in all serum TSH reports with levels ≥ 20 µIU/ml.

These recommendations put forward by the audit team were implemented successfully. Although there were some hitches initially, and the pathway defined was followed rigorously and monitored regularly.

### Conclusion

Newborn screening for congenital hypothyroidism is perceived as a very beneficial publish health program. All developed countries and many developing countries now have established newborn screening programs, at least for CH. Laboratory services for CH newborn screening can be improved by introducing critical results reporting of high TSH levels to the concerned physician. The current audit has effectively improved monitoring of post-analytical practices. Timely informing critical results to the physicians is crucial for patient safety, treatment can be started on time, and irreversible mental retardation can be prevented.

## Limitations

- The limitations of this audit were that thyroid hormones and antibody status of mothers of the neonates was not known. Newborns babies born to mothers with thyroidal illnesses can present with abnormal TSH levels and may be misdiagnosed.
- A single cutoff and not age-related cutoffs were used for screening, adopted from AAP guidelines. Along with serum TSH, FT4 was not performed with the initial sample, and repeat testing results for NTSH of all patients were not known.

## Data Availability

The data set that was used and analyzed in the current study are available from the corresponding author upon reasonable request. The data was not publically available; it was accessed after approval from the section head Chemical Pathology, Dept. of Pathology and Laboratory Medicine Ethical review committee of Aga Khan University.

## Abbreviations

CH: Congenital hypothyroidism
TSH: Thyrod stimulating hormone
NBS: Newborn screening
CLIA: Clinical Laboratory Improvement Amendments
JC: The Joint Commission
CAP: College of American Pathologists
WHO: World Health Organization
AAP: Academy of Pediatrics
FT4: Free thyroxine
ATA: American Thyroid Association
